# Clinical and radiological characterization of fibrous hamartoma of infancy

**DOI:** 10.1590/0100-3984.2015.0085

**Published:** 2017

**Authors:** Vagner Moysés Vilela, Valéria Mota Ribeiro, Jairo Campos Paiva, Diego Demolinari Pires, Lucas Scodeler Santos

**Affiliations:** 1 Universidade Federal de Juiz de Fora (UFJF), Juiz de Fora, MG, Brazil.

**Figure 1:**
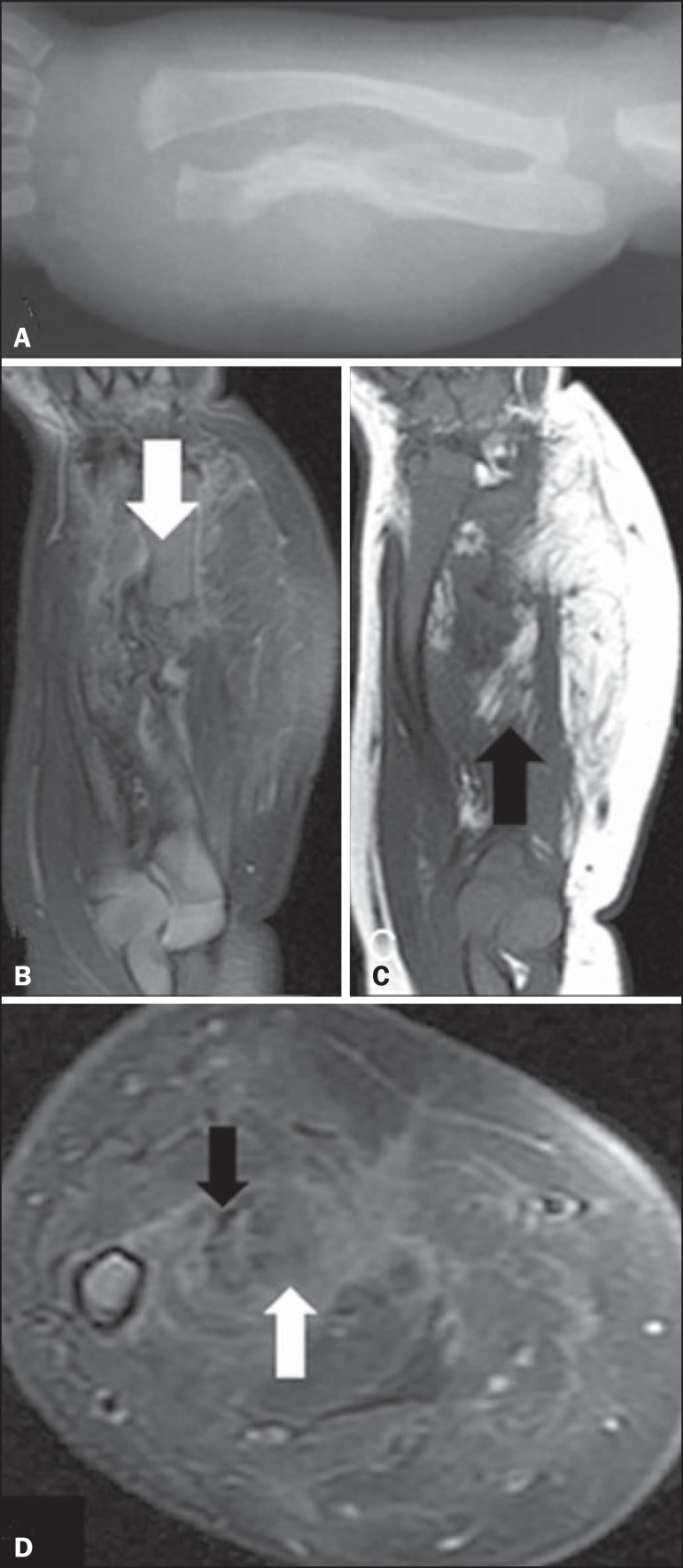
**A:** Forearm X-ray showing fracture associated with ulna irregularity and bowing of the radius, together with increased thickness and density of the soft parts of the forearm. **B:** Fat-saturated, T2-weighted magnetic resonance imaging scan, in the coronal plane, showing discontinuity of the ulna (arrow), the full extent of the lesion, and suppression of the fatty content. **C:** T1-weighted magnetic resonance imaging scan, in the coronal plane, highlighting the lipid content of the lesion (arrow). **D:** Proton-density axial magnetic resonance imaging slice in the region of the fractured ulna showing the contrast uptake by the dense fibrous stroma, the fibrotic streaks (black arrow), and the suppressed signaling of the fat content (white arrow).

Dear Editor,

A newborn male patient presented with suspected obstetric trauma due to increased forearm diameter. An X-ray of the forearm (Figure 1A) showed fracture of the ulna and bowing of the radius, together with increased thickness and density of the adjacent soft tissues. Intravenous contrast-enhanced magnetic resonance imaging (Figures 1B, 1C, and 1D) revealed a heterogeneous, infiltrative tissue formation, with ill-defined and therefore difficult to measure borders, the epicenter of which was in the interosseous membrane of the middle and distal thirds of the forearm. The formation was infiltrating the muscle planes on the volar and dorsal faces of the forearm and was in contact with the vascular-nervous bundles, although there were no signs that it had invaded the bundles. The lesion created discontinuity in the middle third of the ulna and the bowing of the radius. Heterogeneous contrast enhancement was observed, as were lipid material from the lesion and fibrotic streaks.

An incisional biopsy was performed. Histological and immunohistochemical analysis of the biopsy specimen demonstrated positivity for vimentin alpha-actin and for S-100 protein, together with negativity for desmin. On the basis of those findings, the definitive diagnosis of fibrous hamartoma of infancy (FHI) was made.

A benign soft-tissue tumor that typically occurs in the first two years of life^(1)^, FHI was first described in 1956 by Reye^(2)^, who dubbed it subdermal fibromatous tumor of infancy. There have been fewer than 200 cases reported to date, only 8 having been reported in the literature of Latin America; 91% of all cases occurred in the first year of life, 25% having been diagnosed at birth^(3)^.

The differential diagnosis of FHI includes all other soft-tissue tumors. When the tumor is hard and fixed to the deep planes, it is important not to confuse FHI with malignant neoplasms such as juvenile fibromatosis and sarcoma (especially rhabdomyosarcoma and fibrosarcoma, which typically affect young children). Neural tumors (mainly neurofibromas) and vascular tumors should also be excluded^(4,5)^. The identification of fat within the lesion helps narrow down the differential diagnosis, as do patient age and form of presentation. In the appropriate clinical context, a finding of fibrous tissue trabeculae interspersed with fat in an organized pattern is strongly suggestive of FHI^(6)^.

The occurrence of FHI is not related to syndromes or a positive family history^(5)^. Larger lesions typically involve neurovascular structures. Although the tumors are infiltrative, with ill-defined borders and no capsule, the typically do not invade the surrounding bone structures^(5)^.

Reportedly, FHI is painless and its growth is unpredictable. It can grow rapidly in early childhood, its rate of growth slowing after the child has reached five years of age. There have been no reports of spontaneous involution or malignancy^(3)^.

Histopathological examination of an FHI shows compounds of mature adipose tissue interspersed with bands of dense fibrous tissue rich in myofibroblasts and collagen^(5)^. Together with the bands of connective tissue, an FHI presents nests of primitive mesenchyma represented by small, rounded, immature cells, without areas of atypia but occasionally with mitoses, immersed in a myxoid matrix, possibly constituting an anomalous process of tissue maturation.

The preferred treatment for FHI is complete local resection^(6)^, no adjunctive therapies being required. Recurrence after complete resection is uncommon, having been reported in only approximately 10% of cases^(5)^.
